# Estrogenic gper signaling regulates mir144 expression in cancer cells and cancer-associated fibroblasts (cafs)

**DOI:** 10.18632/oncotarget.4117

**Published:** 2015-05-12

**Authors:** Adele Vivacqua, Paola De Marco, Maria Francesca Santolla, Francesca Cirillo, Michele Pellegrino, Maria Luisa Panno, Sergio Abonante, Marcello Maggiolini

**Affiliations:** ^1^ Department of Pharmacy and Health and Nutrition Sciences, University of Calabria, Rende, Italy; ^2^ Regional Hospital, Cosenza, Italy

**Keywords:** CAFs, cancer, estrogens, GPER, miRNAs

## Abstract

MicroRNAs (miRNAs) are small non coding RNA molecules that play a crucial role in several pathophysiological conditions, including cancer. The stimulation of hormone-sensitive tumors by estrogens are mediated by estrogen receptor (ER)α and G protein estrogen receptor (GPER). Previous studies have reported that ERα regulates miRNA expression, while this ability of GPER remains to be elucidated. Here, we demonstrate that in SkBr3 breast cancer and HepG2 hepatocarcinoma cells, 17β-estradiol (E2) and the selective GPER ligand G-1 induce miR144 expression through GPER and the involvement of the PI3K/ERK1/2/Elk1 transduction pathway. Moreover, we show that E2 and G-1 down-regulate through miR144 the onco-suppressor Runx1 and increase cell cycle progression. The capability of E2 and G-1 in triggering the induction of miR144 and the down-regulation of Runx1 was also confirmed in cancer-associated fibroblasts (CAFs) that are main components of the tumor microenvironment driving cancer progression. Further confirming these results, Runx1 protein levels were found decreased in tumor xenografts upon G-1 treatment. On the basis of our findings miR144 and Runx1 may be included among the oncotargets of GPER action. Moreover, the present data provide new insights regarding the ability of estrogens to trigger the GPER/miR144/Runx1 transduction pathway toward the stimulation of cancer progression.

## INTRODUCTION

Several genes encode small functional RNA molecules (of ~22 nucleotides) named microRNAs (miRNAs), which regulate the expression of up to 30% protein coding genes [1]. Primary transcripts (pri-miRNAs) of mature miRNAs, folding into a stable hairpin/stem loop structure, generate miRNA precursors (pre-miRNA). The loop of pre-miRNA is then removed to form a short double-stranded RNA (dsRNA), each single strand can act as a mature miRNA obtained by a special RNaseIII-like endonuclease, called Dicer, which integrates the mature miRNA into the RNA induced silencing complex (RISC) [1]. The RISC complex negatively regulates gene expression through the inhibition of the translation elongation or triggering mRNA destruction, depending on the degree of miRNA complementary with its target [1]. However, it has been also reported that miRNAs may also increase the translation of selected mRNAs in certain cell contexts [2]. Usually, miRNA target sites are located within the 3′ untranslated regions (3′-UTRs) of the mRNAs [3]. The expression of miRNAs displays a cell type and tissue specificity, indicating that miRNAs are closely associated with cell differentiation and development [4, 5]. In addition, miRNAs exert a regulatory role in numerous physiologic and pathologic processes, including many types of tumors [6, 7].

One of the first solid tumors profiled for miRNAs expression is breast cancer, the most common female malignancy in western countries [8]. Among the most significant miRNAs overexpressed in breast carcinoma, miRNA-21 has been shown to mediate cell survival and proliferation by targeting onco-suppressor genes such as PTEN and PDCD4 [9]. Moreover, miRNA21 expression has been associated with advanced clinical stage, lymph node metastasis and poor prognosis [10]. On the contrary, the ectopic expression of miRNA205 in breast cancer cells decreased proliferation and improved the responsiveness to tyrosine kinase inhibitors like gefitinib [11]. miRNAs expression was also related to some biological features of breast carcinoma including the expression of the estrogen receptor (ER) and the progesterone receptor (PR) as well as the tumor grade, stage and invasion [9]. For instance, it was demonstrated that miRNA206 targets directly ERα [9], whereas miRNA221 and miRNA222 may confer a tamoxifen resistance regulating p27 [12] and ERα [13]. In addition, miRNA191 and miRNA425 were reported to be regulated by ERα [14]. As it concerns the hepatocellular carcinoma, the expression levels of miRNA222, miRNA106a, miRNA17/92 and miRNA 135b were associated with tumor differentiation [15, 16], while miRNA125b was correlated with a good survival [17]. The gene encoding human miR144 is located on chromosome 17 and includes a transcriptional unit that encodes also miRNA451 [18].. In erythroid cells, miR144 and miRNA451 form a miRNA cluster which was found decreased in erythroid hyperplasia, ineffective erythropoiesis and mild anemia [19]. However, the role of miR144 in terminal erythropoiesis remains to be determined as its function is still unknown [19]. In solid tumors like breast carcinoma, the expression and action of miR144 have not been fully evaluated. In nasopharyngeal carcinoma, the up-regulation of miR144 led to cell proliferation [20], whereas its reduction was associated with a poor prognosis in patients with colorectal tumors [21]. In human neuroblastoma cells, increased levels of miR144 were found to occur through the nuclear transcription factor AP-1 [22], whereas in rat and human pancreatic islets miR144 expression was found to be up-regulated by 17β estradiol (E2) [23].

Estrogens influence the proliferation, differentiation and physiology of normal and malignant tissues mainly binding to the estrogen receptor (ER)α and ERβ [24]. In recent years, the G protein estrogen receptor namely GPER/GPR30 has been also involved in the estrogen action in numerous types of normal and tumor cells [25, 26]. Estrogenic GPER signalling induces relevant biological responses like gene expression changes, cell proliferation and migration [27-33] by activating several transduction pathways like the epidermal growth factor receptor (EGFR), the mitogen-activated protein kinase/extracellular regulated protein kinase (MAPK/ERK), the phosphatidylinositol 3-kinase/protein kinase B (PI3K/Akt), cyclic adenosine mono-phosphate (cAMP) and phospholipase C (PLC) [25].

In this study, we show that E2 and the selective GPER ligand G-1 up-regulate miR144 expression through the GPER and the involvement of the PI3K/ERK1/2/Elk1 transduction pathway in both breast cancer and hepatocarcinoma cells. Next, we demonstrate that miR144 and its target, namely the onco-suppressor Runx1, are involved in the stimulation of cell cycle progression upon exposure to E2. In addition, we evidence that E2 and G-1 regulate through GPER the expression of miR144 and Runx1 in cancer-associated fibroblasts (CAFs), which are main stimulatory components of the tumor microenvironment. Results similar to those reported above were partially recapitulated in tumor xenografts. Altogether, our data provide new insights into the molecular mechanisms triggered by estrogenic GPER signalling towards miRNA regulation and cancer progression.

## RESULTS

### Transduction mechanisms involved in the up-regulation of miR144 by E2 and G-1

**Figure 1:**
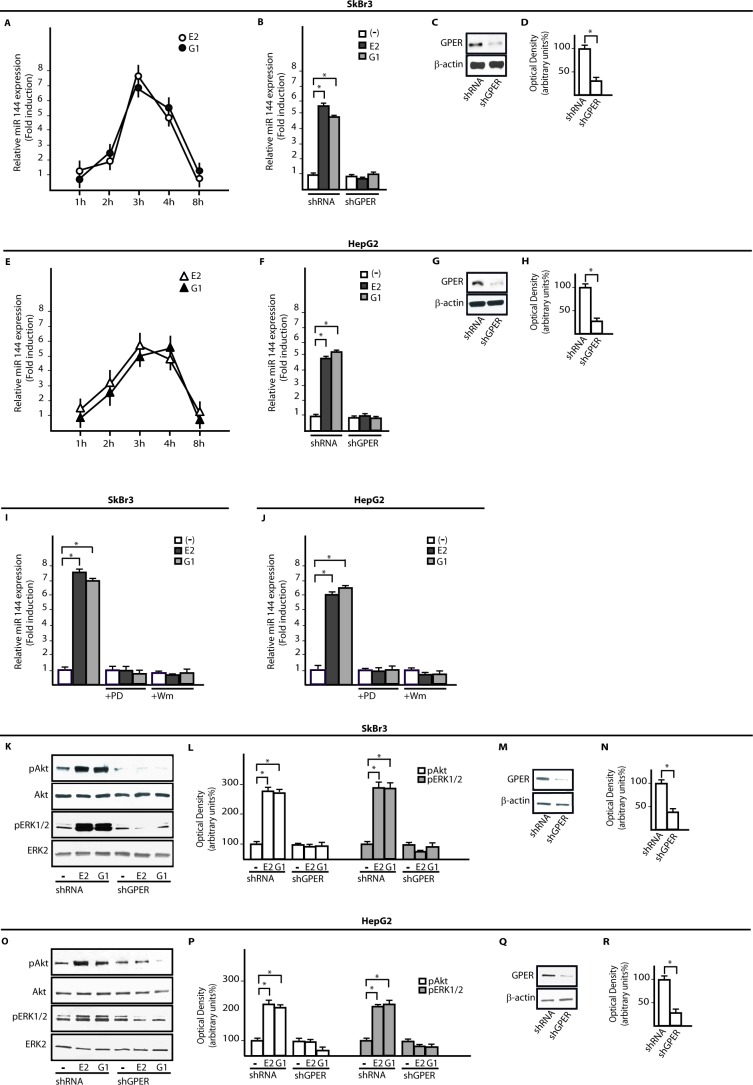
E2 and G-1 up-regulate miR144 expression through GPER and the PI3K-Akt/ERK1/2 transduction pathways miR144 expression in SkBr3 **A.** and HepG2 cells **E.** upon stimulation with 100nM E2 and 100nM G-1, as indicated. Each point is plotted as fold changes of cells receiving treatments respect to cells treated with vehicle and represents the mean ± SD of three independent experiments performed in triplicate. miR144 levels upon a 3h treatment with 100nM E2 and 100nM G-1 in SkBr3 **B.** and HepG2 **F.** cells transfected with shRNA or shGPER. Each column represents the mean ± SD of three independent experiments performed in triplicate. Efficacy of GPER protein silencing in SkBr3 **C.**, **D.** and HepG2 **G.**, **H.** cells as determined by densitometric analysis of GPER expression normalized to β-actin levels. miR144 levels in SkBr3 **I.** and HepG2 **J.** cells treated for 3h with 100nM E2 and 100nM G-1 alone and in presence of 1μM PD98059 (PD) or 1μM Wortmannin (Wm). Each column represents the mean ± SD of three independent experiments performed in triplicate. Akt and ERK1/2 phosphorylation in SkBr3 **K.** and HepG2 **O.** cells transfected with shRNA or shGPER and treated for 5 min with 100nM E2 and 100nM G-1. Densitometric analysis of pAkt and pERK1/2 expression normalized to Akt and ERK2 levels, respectively in SkBr3 **L.** and HepG2 **P.** cells. Efficacy of GPER silencing in SkBr3 **M.**, **N.** and HepG2 **Q.**, **R.** cells as determined by densitometric analysis of GPER expression normalized to β-actin levels. *, *p* < 0.05, for cells receiving treatments *vs* cells treated with vehicle (−).

**Figure 2:**
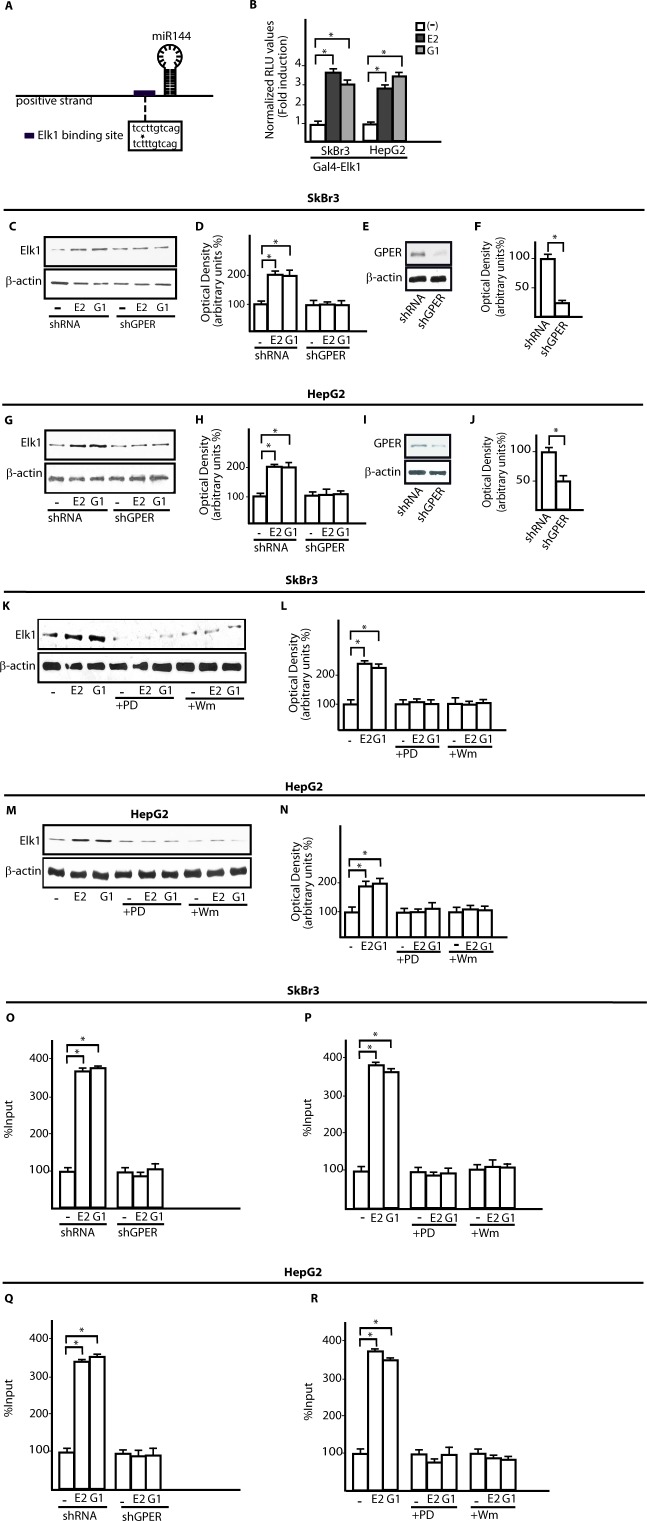
The transcription factor Elk1 is involved in the up-regulation of miR144 by E2 and G-1 **A.** Schematic representation of a putative Elk1 binding site located within the miR144 promoter region. **B.** Luciferase activity in SkBr3 and HepG2 cells transfected with a Gal4-reporter plasmid along with the expression vector Gal4-Elk1 and then treated for 18h with 100nM E2 and 100nM G-1. Each column represents the mean ± SD of three independent experiments performed in triplicate. Elk1 protein levels in SkBr3 **C.** and HepG2 **G.** cells transfected with shRNA or shGPER and treated for 2h with 100nM E2 and 100nM G-1. Densitometric analysis of Elk1 expression normalized to β-actin levels in SkBr3 **D.** and HepG2 **H.** cells. Efficacy of GPER silencing in SkBr3 **E.**, **F.** and HepG2 cells. **I.**, **J.** as determined by densitometric analysis of GPER expression normalized to β-actin levels. Elk1 protein levels in SkBr3 **K.** and HepG2 **M.** cells treated for 2h with 100nM E2 and 100nM G-1 alone or in the presence of 1μM PD98059 (PD) and 1μM Wortmannin (Wm). Densitometric analysis of Elk1 expression normalized to β-actin levels in SkBr3 **L.** and HepG2 **N.** cells. Recruitment of Elk1 to the miR144 promoter sequence in SkBr3 **O.** and HepG2 **Q.** cells transfected with shRNA or shGPER and treated for 2h with 100nM E2 and 100nM G-1. Recruitment of Elk1 to the miR144 promoter sequence in SkBr3 **P.** and HepG2 **R.** cells treated for 2h with 100nM E2 and 100nM G-1 alone and in the presence of 1μM PD98059 (PD) or 1μM Wortmannin (Wm). Each column represents the mean ± SD of three independent experiments performed in triplicate. *, *p* < 0.05, for cells receiving treatments *vs* cells treated with vehicle (−).

On the basis of previous data indicating that E2 regulates certain miRNAs as miR144 [23], ER-negative SkBr3 breast cancer and HepG2 hepatocarcinoma cells (Supplementary Figure 1) [27, 29] were treated with 100nM E2 and 100nM G-1 to assess miR144 expression by real-time PCR. Worthy, the increase of miR144 levels induced by both compounds (Figure 1A, 1E) was no longer evident silencing GPER in SkBr3 and HepG2 cells (Figure 1B-1D and 1F-1H). Then, we ascertained that two main transduction pathways involved in GPER signalling, like ERK1/2 and PI3K-Akt [25], contribute to the up-regulation of miR144 by 100nM E2 and 100nM G-1 as this response was abrogated in the presence of 1μM MEK inhibitor PD or 1μM PI3K inhibitor Wm (Figure 1I, 1J). Next, a rapid Akt and ERK1/2 phosphorylation (5 min) triggered by 100nM E2 and 100nM G-1 was abolished silencing GPER expression (Figure 1K-1R). To further investigate the mechanisms involved in the transcription of miR144, we analyzed its ~-1kb promoter region (http://www.ncbi.nlm.nih.gov; http://www.generegulation.com) identifying a putative Elk1 binding site (Figure 2A). Hence, we transfected cells with a Gal4-Elk1 construct together with the reporter gene Gal4-Luc, detecting a substantial luciferase activity upon treatment with 100nM E2 and 100nM G-1 (Figure 2B). In accordance with these results, the protein levels of Elk1 were up-regulated treating cells for 2h with 100nM E2 and 100nM G-1, however this response was prevented silencing GPER (Figure 2C-2J) or in the presence of 1μM PD or 1μM Wm (Figure 2K-2N). Thereafter, in both SkBr3 and HepG2 cells ChIP analysis revealed that Elk1 is recruited within the miR144 promoter region upon a 2h exposure to 100nM E2 and 100nM G-1, however this recruitment was no longer evident silencing GPER (Figure 2O, 2Q) or treating cells with 1μM PD or 1μM Wm (Figure 2P, 2R). Altogether, these findings indicate that the up-regulation of miR144 induced by E2 and G-1 involves GPER and a transduction network in SkBr3 and HepG2 cells.

### miR144 regulates the onco-suppressor Runx1

**Figure 3:**
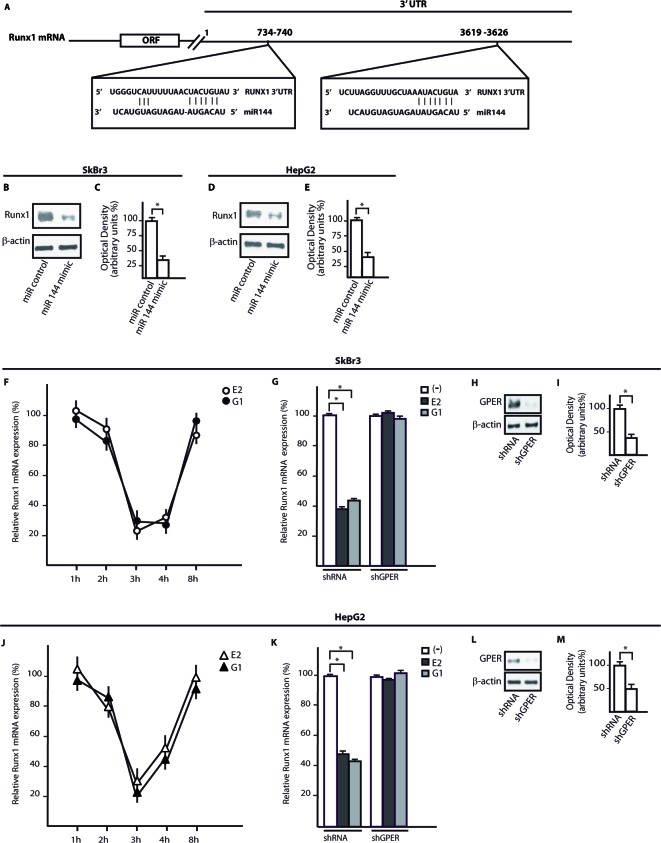
Runx1 is a target gene of mir144 and its expression is down-regulated by E2 and G-1 through GPER **A.** Alignment between the miR144 sequence and the 3′-UTR mRNA region of Runx1. Runx1 protein levels in SkBr3 **B.** and HepG2 **D.** cells transfected with a mimic miR144 sequence. Densitometric analysis of the Runx1 expression normalized to β-actin levels in SkBr3 **C.** and HepG2 **E.** cells. The mRNA levels of Runx1 are down-regulated in SkBr3 **F.** and HepG2 **J.** cells treated with 100nM E2 and 100nM G-1, as indicated. Each point is plotted as fold changes of cells receiving treatments respect to cells treated with vehicle and represents the mean ± SD of three independent experiments performed in triplicate. Runx1 mRNA expression in SkBr3 **G.** and HepG2 **K.** cells transfected with shRNA or shGPER and treated for 3h with 100 nM E2 and 100nM G-1. Each column represents the mean ± SD of three independent experiments performed in triplicate. Efficacy of GPER silencing in SkBr3 **H.**, **I.** and HepG2 **L.**, **M.** cells as determined by densitometric analysis of GPER expression normalized to β-actin levels. *, *p* < 0.05, for cells receiving treatments *vs* cells treated with vehicle (−).

**Figure 4:**
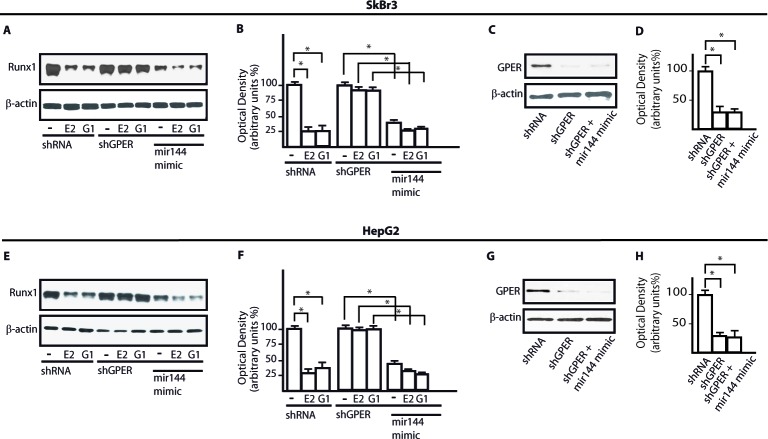
GPER and miR144 are involved in the down-regulation of Runx1 protein expression induced by E2 and G-1 Runx1 protein expression in SkBr3 **A.** and HepG2 **E.** cells transfected with shRNA, shGPER alone and in combination with a mimic miR144 sequence and treated for 3h with 100nM E2 and 100nM G-1. Densitometric analysis of the Runx1 expression normalized to β-actin levels in SkBr3 **B.** and HepG2 **F.** cells. Efficacy of GPER silencing in SkBr3 **C.**, **D.** and HepG2 **G.**, **H.** cells as determined by densitometric analysis of GPER expression normalized to β-actin levels. *, *p* < 0.05, for cells receiving treatments *vs* cells treated with vehicle (−).

**Figure 5:**
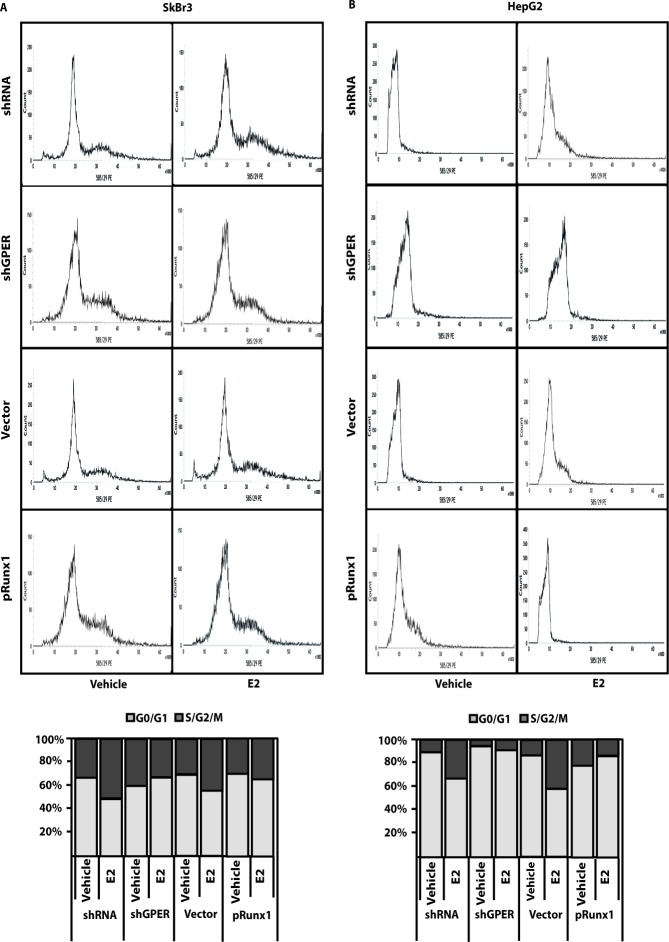
GPER silencing and Runx1 over-expression prevent cell-cycle progression induced by E2 Cell cycle analysis in SkBr3 **A.** and HepG2 **B.** cells transfected for 24h with shRNA, shGPER and plasmids encoding for a vector or Runx1 (pRunx1), then treated for 24h with 100nM E2. The histograms show the percentage of cells in G0/G1 and S/G2/M phases of the cell cycle. Values represent the mean ± SD of three independent experiments.

Using available bioinformatics algorithms (http://www.microrna.org; http://www.miRNAbase.org; http://www.targetscan.org), we determined that miR144 may regulate Runx1 on the basis of the putative target sequences located within the 3′-UTR Runx1 region (Figure 3A). A further bioinformatic analysis (http://ecrbrowser.dcode.org) revealed that the putative MiRNA Responsive Elements (MREs) to miR144 located within the 3′-UTR Runx1 sequence are conserved in Vertebrates (Supplementary Tab. 1). Taking into account the aforementioned data, we transfected SkBr3 and HepG2 cells with a miR144 mimic sequence which triggered a decrease of Runx1 protein levels (Figure 3B-3E). Thereafter, we analyzed the Runx1 mRNA expression in SkBr3 and HepG2 cells upon treatment with 100nM E2 and 100nM G-1, as these ligands induce the up-regulation of miR144. Interestingly, the mRNA expression of Runx1 decreased in a time-dependent manner (Figure 3F, 3J), however this response was no longer evident silencing GPER (Figure 3G-3I and Figure 3K-3M). Further confirming these results, the Runx1 protein levels were lowered treating SkBr3 and HepG2 cells for 3h with 100nM E2 and 100nM G-1 (Figure 4A, 4B and 4E, 4F). Worthy, the decrease of Runx1 protein was prevented transfecting cells with the shGPER construct, but not co-transfecting the miR144 mimic sequence (Figure 4A-4H). Biologically, the treatment for 24h with 100nM E2 induced in SkBr3 and HepG2 the progression of cell cycle (Figure 5A, 5B), however this response was abolished silencing GPER or transfecting cells with an expression vector of Runx1 (pRunx1) (Figure 5A, 5B). Taken together, our results suggest that in SkBr3 and HepG2 cancer cells E2 and G-1 through GPER induce miR144 expression which negatively regulates the levels of its target gene Runx1.

### E2 and G-1 through GPER up-regulate miR144 expression which lowers the levels of Runx1 in CAFs

**Figure 6:**
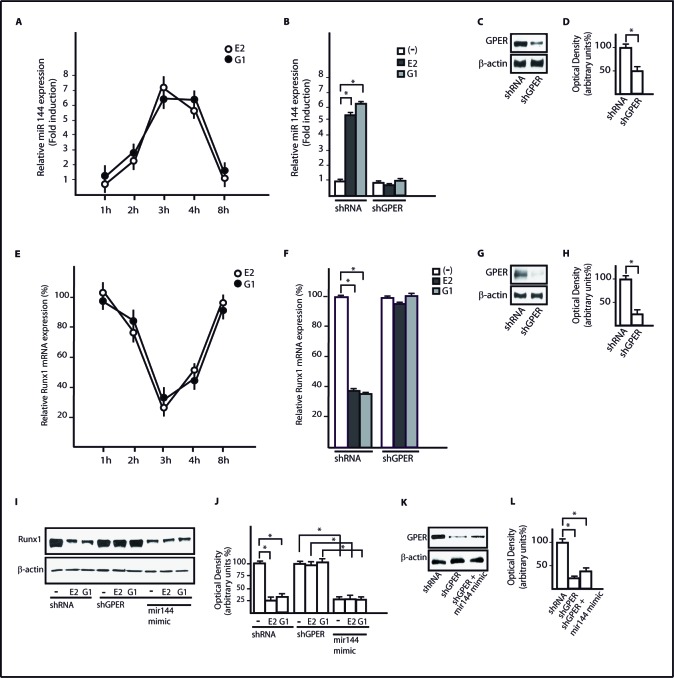
GPER mediates the up-regulation of miR144 and the reduction of Runx1 induced by E2 and G-1 in CAFs **A.** miR144 expression in CAFs upon stimulation with 100nM E2 and 100nM G-1, as indicated. Each point is plotted as fold changes of cells receiving treatments respect to cells treated with vehicle and represents the mean ± SD of three independent experiments performed in triplicate. **B.** miR144 expression in CAFs transfected with a shRNA or shGPER and treated for 3h with 100nM E2 and 100nM G-1. Each column represents the mean ± SD of three independent experiments performed in triplicate. **C.**, **D.** Efficacy of GPER silencing in CAFs as determined by densitometric analysis of GPER expression normalized to β-actin levels. **E.** Runx1 mRNA expression in CAFs upon treatment with 100nM E2 and 100nM G-1, as indicated. Each point is plotted as fold changes of cells receiving treatments respect to cells treated with vehicle and represents the mean ± SD of three independent experiments performed in triplicate. **F.** Runx1 mRNA expression in CAFs transfected with shRNA or shGPER and treated for 3h with 100nM E2 and 100nM G-1. Each column represents the mean ± SD of three independent experiments performed in triplicate. **G.**, **H.** Efficacy of GPER silencing in CAFs as determined by densitometric analysis of GPER expression normalized to β-actin levels. **I.** Runx1 protein expression in CAFs transfected with shRNA, shGPER alone and in combination with a mimic miR144 sequence, then treated for 3h with 100nM E2 and 100nM G-1. **J.** Densitometric analysis of Runx1 protein expression normalized to β-actin levels. **K.**, **L.** Efficacy of GPER silencing in CAFs as determined by densitometric analysis of GPER expression normalized to β-actin levels. *, *p* < 0.05, for cells receiving treatments *vs* cells treated with vehicle (−).

Considering the relevant contribution of the microenvironment to cancer cell growth and invasiveness [34], we examined the regulation of miR144 and Runx1 expression in CAFs, which are key components of the tumor microenvironment. We found that the treatments with 100nM E2 and 100nM G-1 increase miR144 expression (Figure 6A), however this response was prevented silencing GPER expression (Figure 6B-6D). Worthy, the down-regulation of Runx1 mRNA and protein levels observed in CAFs treated whit 100nM E2 and 100nM G-1 was no longer evident knocking down GPER or transfecting the miR144 mimic sequence (Figure 6E-6L). Altogether, these results indicate that GPER mediates the regulation of miR144 and Runx1 by E2 and G-1 also in CAFs, which play relevant stimulatory effects through a functional interaction with cancer cells [34].

### G-1 down-regulates the expression of Runx1 *in vivo*

**Figure 7:**
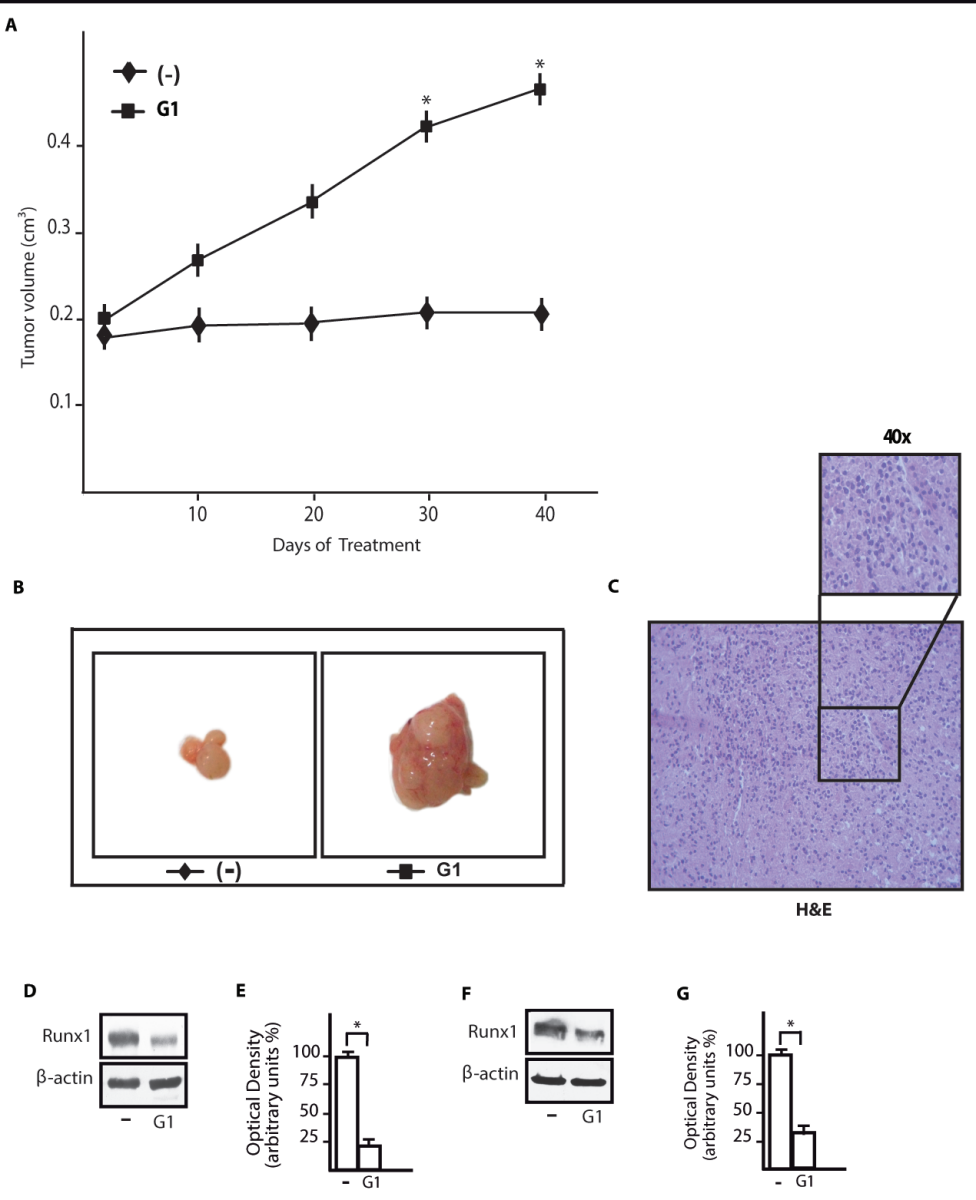
G-1 down-regulates Runx1 expression in tumor xenografts **A.** Tumor volume from SkBr3 xenografts implanted in female athymic nude mice treated for 40 days with vehicle (−) and G-1 (0,5 mg/Kg/die), as indicated. *, *p* < 0.05, for animals treated with G-1 *vs* animals treated with vehicle. **B.** Representative images of SkBr3 xenograft tumors at 40 days, scale bar: 0.3 cm. **C.** Representative tumor section from mice at 40 days, formalin fixed, paraffin embedded, sectioned and stained with hematoxylin and eosin Y (H&E). **D.** Runx1 protein expression in tumor homogenates from SkBr3 xenografts treated as described in **A.**. **E.** Densitometric analysis of Runx1 protein expression normalized to β-actin levels. **F.** Runx1 protein expression in cultured SkBr3 cells obtained from tumor xenografts and treated for 3h with 100nM G-1. **G.** Densitometric analysis of Runx1 protein expression normalized to β-actin levels. * *p* < 0.05, for cells receiving treatments *vs* cells treated with vehicle (−).

In order to further evaluate the aforementioned response of Runx1 upon ligand activation of GPER, we turned to an *in vivo* experimental model. Hence, SkBr3 cells were injected into the intrascapular region of female nude mice and tumor growth was monitored upon the administration of vehicle or 0.5mg/kg/die G-1. This treatment was well tolerated because no change in body weight or in food and water consumption was observed, along with no evidence of reduced motor function. In addition, after sacrifice no significant differences in the mean weights or histological features of major organs (for instance liver, lung, spleen and kidney) were observed between vehicle-treated mice and those receiving the treatment, indicating a lack of toxic effects at the given dose. A significant increase in tumor volume was observed starting from 30 days of treatment with G-1 (Figure 7A) and after 40 days the mice were sacrificed (a representative tumor is shown in Figure 7B). Histological examination of SkBr3 xenografts by hematoxylin and eosin staining revealed that samples were mostly composed of tumor epithelial cells (Figure 7C). In tumor homogenates obtained from G-1 stimulated mice we detected an increased expression of the proliferative marker Ki67 respect to mice treated with vehicle (Supplementary Figure 2). In addition, in tumor homogenates from G-1 treated mice we found a decrease of Runx1 protein expression respect to vehicle treated mice (Figure 7D, 7E). Culturing SkBr3 cells obtained from tumor xenografts, we further confirmed the down-regulation of Runx1 protein expression upon treatment with 100nM G-1 for 3h (Figure 7F, 7G). Altogether, these data suggest that G-1 stimulates the growth of SkBr3 tumor xenografts and reduces Runx1 protein expression also *in vivo*.

## DISCUSSION

In the present study, we show that in SkBr3 breast cancer and HepG2 hepatocarcinoma cells E2 and G-1 induce the expression of miR144 and down-regulate the levels of Runx1 through GPER and the involvement of the PI3K/ERK1/2/Elk1 transduction pathway. Moreover, we demonstrate that E2 and G-1 modulate the expression of miR144 and Runx1 also in main components of the tumor microenvironment like CAFs. Biologically, the increase in S/G2/M phases of the cell cycle upon exposure to E2 was no longer evident silencing GPER or overexpressing Runx1. Worthy, G-1 triggered *in vivo* tumor growth and decreased Runx1 expression in SkBr3 xenografts. Altogether, our findings provide new insights into the potential of estrogenic GPER signalling to mediate cancer progression through the involvement of miR144 and Runx1 in both cancer cells and CAFs. In this regard, our data highlight additional mechanisms by which tumor cells and the microenvironment cooperate toward worse cancer features.

Numerous studies have suggested in the last years that every cellular process is likely regulated by miRNAs and an aberrant miRNA expression may be a hallmark of several diseases, including cancer (4). However, it remains to be fully elucidated the expression and function of various miRNAs in the different types of tumors. For instance, there is a growing interest on the role of miR144 in tumorigenesis and cancer therapy. Previous studies have reported a down-regulation of miR144 in malignancies like osteosarcoma and mesothelioma, suggesting that miR144 might be considered as a potential tumor suppressor [35, 36]. An inverse correlation between the levels of miR144 and the development of gastric and pancreatic cancers has been also reported [37]. However, other investigations have demonstrated an increase of miR144 levels in colorectal [38] and in nasopharyngeal carcinoma [20]. In addition, the inhibition of miR144 led to a decreased proliferation in HeLa cells [39]. In this context, our data indicate that estrogens induce miR144 expression, as previously observed in a different model system [23]. Besides, the present study demonstrates that the E2-stimulated miR144 expression may elicit oncogenic effects in SkBr3 and HepG2 cells, although a forced overexpression of miR144 has been reported to suppress proliferation, migration and invasion in hepatocellular carcinoma HCC cells [40]. These controversial results may rely on the different experimental conditions, including the cell types used and the action of the endogenous miR144 evaluated in our investigation. Anyway, these findings address the need to further ascertain the function exerted by miR144 in tumorigenesis and cancer progression.

In order to better understand the biological relevance of the increased miR144 expression upon exposure to E2 and G-1 in cells used, we identified the tumor suppressor Runx1 (known also as AML1) as a miR144 target gene through a bioinformatics analysis. Runx1 is a member of the mammalian Runx family encoding for the transcription factors Runx1, 2 and 3 that may function as tumor suppressors binding to specific DNA sequences namely PEBP2 sites (TGT/CGGT) [41]. Actually, Runx family members can also act as oncogenes, depending on diverse factors including the cellular context [42]. Runx1 is the predominant Runx family member expressed in breast epithelial cells as well as in the basal and the luminal cell layer [43]. Recurrent mutations of Runx1 have been detected in breast cancer, suggesting that aberrations in Runx-mediated cell differentiation may drive the development of this malignancy [44]. In addition, Runx1 expression was found less abundant in breast cancer cells compared to normal breast epithelial cells and its levels have been reported to decrease progressively with the tumor aggressiveness [45]. Experimental Ras-mediated transformation of basal-like MCF10A cells was associated with the loss of Runx1 [46] and the silencing of its expression in the same cells led to the formation of abnormal, hyperproliferative acinar structures in 3D cultures [47]. In accordance to previous studies suggesting a tumor-suppressor role of Runx1 [41], we found that the increased number of SkBr3 and HepG2 cells in S/G2/M phases upon exposure to E2 is no longer evident in cells overexpressing Runx1. Further confirming these results, the treatment with G-1 decreased the expression of Runx1 and induced growth effects in SkBr3 xenografts.

Estrogens can act through the classical ER and GPER in triggering important biological responses, including the progression of several types of tumors [24, 25]. Although the involvement of ERα in the regulation of miRNAs by estrogens has been well described [48], the possible role of GPER on the expression of miRNAs has been reported in a few studies [23, 49]. Thus, our results provide further insights into the potential of GPER to mediate the estrogenic regulation of miRNAs in cancer cells like SkBr3 and HepG2 as well as in CAFs that exert a stimulatory action within the tumor microenvironment. In particular, the present data indicate the potential of GPER to modulate miR144 and Runx1 expression upon estrogen exposure towards cancer progression. In addition, the GPER-mediated responses through miR144 and Runx1 in CAFs extend the current knowledge on the critical interactions between cancer cells and important components of the surrounding stroma toward tumor development and metastasis. Our results suggest also that the GPER/miR144/Runx1 signaling pathway may represent a further oncotarget to be considered in innovative therapeutic approaches in estrogen-sensitive tumors.

## MATERIALS AND METHODS

### Bioinformatic tools

The putative promoter sequences of primary miR144 and the 3′-UTR of Runx1 were retrieved from the National Center for Biotechnology Information (NCBI) (http://www.ncbi.nlm.nih.gov). Prediction of transcription factors for primary miR144 was performed using TRANSFAC (http://www.generegulation.com) site. miR144 targets genes were identified using miRanda (http://www.microrna.org/), miRNAbase (http://www.miRNAbase.org) and Targetscan (http://www.targetscan.org) sites. Sequences conservation was analyzed with the Evolutionary Conserved Region Browser (http://ecrbrowser.dcode.org).

### Reagents

17β-estradiol (E2) was purchased from Sigma-Aldrich Corp. (Milan, Italy). MEK inhibitor PD98059 (PD) and PI3K inhibitor Wortmannin (Wm) were bought from Calbiochem (Milan, Italy). 1-[4-(-6-bromobenzol[1,3]diodo-5yl)-3a,4,5,9b-tetrahidro3H-cyclopenta[c]quinolin-8yl]-ethanone (G-1) was obtained from Biomol Reaserch Laboratories, Inc. (DBA, Milan, Italy). All compounds were solubilized in DMSO, except E2, which was dissolved in ethanol.

### Cell cultures

SkBr3 and MCF-7 breast cancer, HepG2 hepatocarcinoma and LNCaP prostate cancer cells were obtained by ATCC (Manassas, USA) and used less than six months after revival. SkBr3 and LNCaP were maintained in RPMI-1640 without phenol red, MCF-7 and HepG2 cells were maintained in DMEM medium, with a supplement of 10% fetal bovine serum (FBS) and 100 μg/ml of penicillin/streptomycin (Gibco, Life Technologies, Milan, Italy). CAFs were extracted from breast cancer tissues as previously described [50]. Signed informed consent from all the patients was obtained and all samples were collected, identified and used in accordance with approval by the Institutional Ethical Committee Board (Regional Hospital of Cosenza, Italy). CAFs were cultured in a mixture of MEDIUM 199 and HAM'S F-12 (1:1) supplemented with 10% FBS and 100 μg/ml of penicillin/streptomycin (Gibco, Life Technologies, Milan, Italy). All cells were switched to medium without serum the day before experimental analysis.

### Plasmid and transfections

Short hairpin construct against human GPER (shGPER) and short hairpin scrambled (shRNA) were obtained and used as previously described [51]. In brief, they were generated in the lentiviral expression vector pLKO.1 purchased by Euroclone (Milan, Italy). The targeting strand generated from the shGPER construct was 5′-CGCTCCCTGCAAGCAGTCTTT-3′. The efficacy of GPER silencing was determined by real-time PCR (data not shown) and immunoblotting (see results section). The expression vector Gal4-Elk1 and the reporter plasmid Gal4-Luc were obtained as indicated previously [52]. The mimic miR144 and negative control (NC) sequences were purchased from Ambion (Life Technologies, Milan, Italy). Addgene plasmid 12426: pCMV5-AML1B/Runx1 (pRunx1) (Addgene, Cambridge, MA, USA), used for Runx1 overexpression, was obtained from Dr. Scott Hiebert [53]. Transfection assays were performed using the X-tremeGene9 Transfection Reagent (Roche Diagnostics, Milan, Italy) according to the manufacturer's instructions.

### RNA extraction and real time-PCR

Cells were maintained in regular growth medium and then switched to medium lacking serum for 24h, before to add 100nM E2 and 100nM G-1 for the times indicated. miRNAs and the RNA fraction deprived of small RNA were extracted from cultured cells using miRVana Isolation Kit (Ambion, Life Technologies, Milan, Italy) according to the manufacturer's recommendations. The RNA concentrations were determined with Gene5 2.01 Software in Synergy H1 Hybrid Multi-Mode Microplate Reader (BioTek, AHSI, Milan Italy). cDNA was synthesized from 5ng of total miRNA using the TaqMan microRNA Reverse Transcription Kit (Applied Biosystems, Life Technologies, Milan, Italy) and the expression levels of miR144 were quantified by TaqMan microRNA Assay Kit (Applied Biosystems, Life Technologies, Milan, Italy). Real-time PCR analysis for mature miR144 was performed using the primers for the internal control RNU6B 5′ - TGACACGCAAATTCGTGAAGCGTTCCATATTTTT - 3′ (forward) and miR144 5′-UACAGUAUAGAUGAUGUACU-3′ (forward). In order to measure the mRNA levels of Runx1, 2 μg of RNA fraction deprived of small RNA were reversely transcribed using the murine leukaemia virus reverse transcriptase (Life Technologies, Milan, Italy), following the protocol provided by the manufacturer. The quantitative PCR was performed using SYBR Green PCR Master Mix (Applied Biosystems, Life Technologies, Milan, Italy). Specific primers for *Actin*, which was used as internal control, *ERα, ERβ, GPER* and *Runx1* genes were designed using Primer Express version 2.0 software (Applied Biosystems Inc, Milano, Italy). The sequences were as follows: Actin Fwd: 5′-AAGCCACCCCACTTCTCTCTAA-3′ and Rev: 5′-CACCTCCCCTGTGTGGACTT-3′ (reverse); ERα Fwd: 5′-AGAGGGCATGGTGGAGATCTT-3′ and Rev: 5′-CAAACTCCTCTCCCTGCAGATT-3′; ERβ Fwd: 5′-GACCACAAGCCCAAATGTGTT-3′ and Rev: 5′-ACTGGCGATGGACCACTAAA-3′; GPER Fwd: 5′-ACACACCTGGGTGGACACAA-3′ and Rev: 5′-GGAGCCAGAAGCCACATCTG-3′; Runx1 Fwd: 5′-GCTGGAAAGCAAACAGGAAGA-3′ and Rev: 5′-CAGCATTGCTAAATCAGAAGCATT-3′. All experiments were performed in triplicate using StepOne^TM^ Real-Time PCR Detection System (Applied Biosystems, Life Technologies, Milan, Italy). The data were normalized to the geometric mean of housekeeping gene to control the variability into expression levels and fold changes were calculated by relative quantification.

### Western blotting

Tumor homogenates, obtained from nude mice were processed as previous described [27]. Cells were maintained in medium without serum for 24h, exposed to ligands as indicated and then lysed in RIPA buffer containing a mixture of protease inhibitors. Equal amounts of protein extract were resolved on SDS-polyacrylamide gel, transferred to a nitrocellulose membrane (Amersham Biosciences, Italy), probed overnight at 4°C with antibodies against phosphorylated ERK1/2 (E-4), ERK2 (C-14), phosphorylated Akt 1/2/3 (ser 473), Akt (H-136), Elk1 (E-5), Runx1 (A-2), GPER (N-15), β-Actin (AC-15), Ki67 (H-300) ERα (F-10) (Santa Cruz Biotechnology, DBA, Italy) and ERβ (Serotec), and then revealed using the ECL^TM^ Western Blotting Analysis System (GE Healthcare, Italy).

### Luciferase assays

Cells were plated in regular growth medium and transferred in that without serum on the day of transfection using a mixture containing Gal4-Luc (500ng/well), Gal4-Elk1 (100ng/well) and pRL-TK (10ng/well). After 6h, cells were treated whit 100nM E2 and 100nM G-1 before to incubate for 18h. Luciferase activity was measured with the Dual Luciferase kit (Promega, Italy) according to the manufacturer's instructions. Firefly luciferase values were normalized to the internal transfection control provided by the Renilla luciferase activity. The normalized relative light unit (RLU) values obtained from cells stimulated with vehicle (−) were set as 1-fold induction upon which the activity induced by treatments was calculated.

### Chromatin immunoprecipitation (ChIP) assays

The day before ChIP analysis, cells were shifted to medium lacking serum and then transfected for 48h with 5 μg of the indicated constructs or pre-treated for 30min with the inhibitors PD and WM. Next, the cells were stimulated for 2h with 100nM E2 and 100nM G-1. ChIP assay was performed as previously described [28]. In brief, the immune cleared chromatin was immunoprecipitated with anti Elk1 or normal mouse serum IgG (Santa Cruz Biotecnology, DBA), used as negative control. A 4μl volume of each sample and input DNA were used as template to amplify, by real-time PCR (Applied Biosystems, Italy), a region containing an Elk1 site located within the positive strand of miR144 promoter. The primer sequences were: 5′-TGCCCTGGCAGTCAGTAGGT-3′ (forward) and 5′-ACAGTGCTTTTCAAGCCATG-3′ (reverse). The data were normalized with respect to input. The relative antibody-bound fractions were normalized to a calibrator that was chosen to be the sample treated with vehicle (−).

### Cell cycle analysis

To analyse cell cycle distribution, cells were cultured in regular medium in 6 well plates and shifted in medium containing 2.5% charcoal-stripped FBS when cells were 70% confluents. Next, 5 μg of shRNA or shGPER or pRunx1 were added to cells using X-treamGene9 reagent (Roche Diagnostics, Milan, Italy). After 24h, 100nM E2 were put in the medium for additional 24h. Cells were pelleted, once washed with phosphate buffered saline and fixed in 50% methanol overnight at −20°C, before to stain with a solution containing 50 μg/ml propidium iodide in 1xPBS (PI), 20U/ml RNAse-A and 0.1% Triton (Sigma-Aldrich, Milan, Italy). Cells were analyzed for DNA content by Fluorescence-Activated Cell Sorting (FACS Jazz, BD, Milan, Italy). The proportion of the cells in G0/G1 and S/G2-M phases was estimated each as a percentage of the total events (10,000 cells).

### *In vivo* studies

Animal care, death, and experiments were done in accordance with the U.S. National Institutes of Health Guide for the Care and Use of Laboratory Animals (NIH Publication No. 85-23, revised 1996) and in accordance with the Italian law (DL 116, January 27, 1992). The project was approved by the local ethical committee. Female 45-day-old athymic nude mice (nu/nu Swiss; Harlan Laboratories) were maintained in a sterile environment. At day 0, 8.0 × 10^6^ per mouse of SkBr3 cells exponentially growing, were inoculated subcutaneously in 100μl of Matrigel (Cultrex; Trevigen Inc.). After about 1 week, when the tumors reached average ~0.15 cm^3^, mice were randomized and divided into two groups, which was then treated by intramuscular injection for 40 days in according to treatments used. The first group of mice (*n* = 7) was treated daily with 100μl of vehicle (0.9% NaCl with 0.1% albumin and 0.1% Tween-20; Sigma-Aldrich), whereas the second group of mice (*n* = 7) was treated with 100μl G-1 (0.5 mg/kg/die). G-1 was dissolved in DMSO at 1 mg/ml. For treatments, 6.2 μl of G-1 were added to 93.8μl of vehicle. SkBr3 xenograft tumors were measured using digital vernier calipers and tumor volume calculated using the formula [sagittal dimension (mm) x cross dimension (mm)] 2/2, expressed in cm^3^. At day 40, all animals were sacrificed following the standard protocols and tumors were dissected from the neighboring connective tissue. Specimens of tumors were frozen in nitrogen and stored at −80°C, the remaining tumor tissues of each sample were fixed in 4% paraformaldehyde and embedded in paraffin for the histologic analyses.

### Histologic analysis

Morphologic analyses were carried out on formalin-fixed, paraffin-embedded sections of tumor xenografts, which were cut at 5μm and allowed to air dry. Deparaffinized, rehydrated sections were stained for 6 min with hematoxylin (Bio-Optica, Milan, Italy), washed in running tap water and counterstained with eosin Y (Bio-Optica, Milan, Italy). Sections were then dehydrated, cleared with xylene, and mounted with resinous mounting medium. Tumor sections were also immunolabeled with Ki67, considered as a cell proliferation marker.

### Statistical analysis

Statistical analysis was performed using ANOVA followed by Newman-Keuls' testing to determine differences in means. *p* < 0.05 was considered as statistically significant. Statistical comparisons for *in vivo* studies were obtained using the Wilcoxon-Mann-Whitney test. *p* < 0.05 was considered statistically significant.

## SUPPLEMENTARY MATERIAL FIGURES AND TABLE


