# Supplementary material for: Estrogenic gper signaling regulates mir144 expression in cancer cells and cancer-associated fibroblasts (cafs)
Source: Oncotarget. 2015 May 12;6(18):16573–87. doi: 10.18632/oncotarget.4117 (PMC4599290; doi:10.18632/oncotarget.4117)
Supplement: Supplementary file 1 [file oncotarget-06-16573-s001.pdf]

Estrogenic gper signaling regulates mir144 expression in cancer cells and cancer-associated fibroblasts (cafs)

Supplementary Material

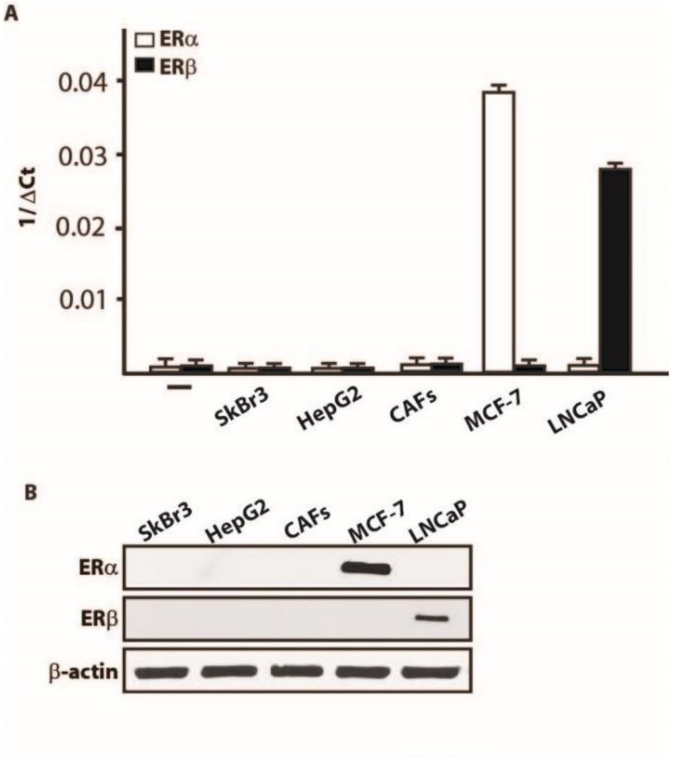

Supplementary Figure 1

Supplementary Figure 1. ERα and ERβ mRNA (A) and protein (B) expression in SkBr3, HepG2, CAFs, MCF-7 and LNCaP cells, as evaluated respectively by real-time PCR and immunoblotting. In RNA experiments, PCR amplification in absence of cDNA was used as a control (-) and each data point represents the mean ± SD of three independent experiments performed in triplicate. In immunoblot assays β-actin was used as loading control.

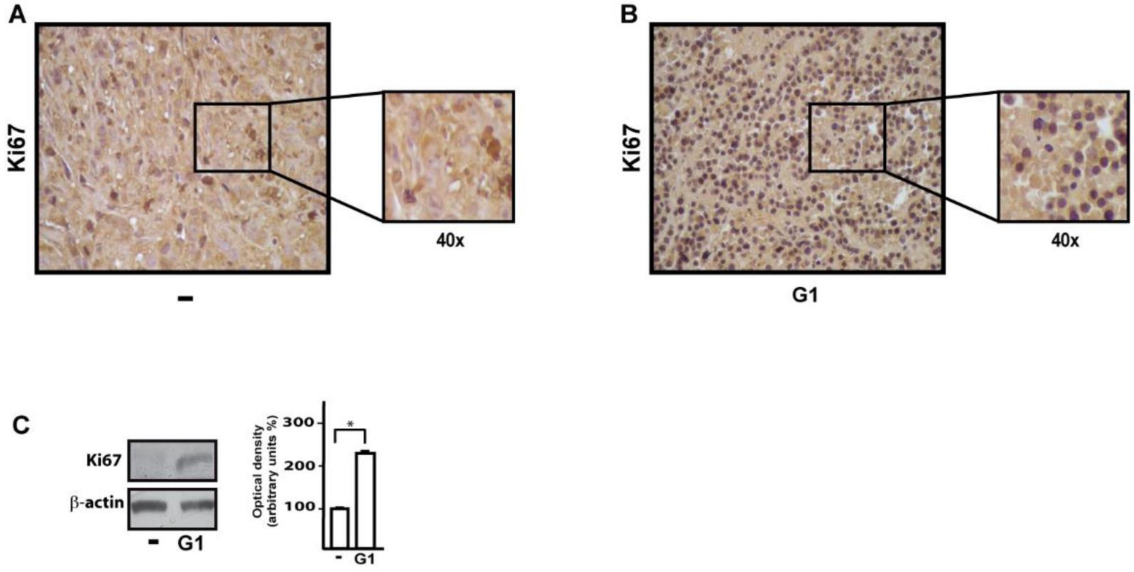

Supplementary Figure 2

Supplementary Figure 2. Evaluation of cell proliferation marker Ki67 in SkBr3 tumor xenografts. Representative images from tumors of mice treated for 40 days with vehicle (A) or G-1 (0,5 mg/kg/die) (B), as indicated. Tumors were formalin fixed, paraffin embedded, sectioned and incubated with a rabbit monoclonal antibody directed against Ki67 that appears as brown staining. (C) Ki67 protein levels in tumor homogenates from SkBr3 xenografts treated as reported above, side panel shows densitometric analysis of the blot normalized to β-actin. *, $p < 0.05$ for G-1 treated animals vs vehicle treated animals.

Species	Position (bp) 734-740	Position (bp) 3619-3626
Hsa	-------------AAC**UACUGU**AUUCCCACAA-----------------------------------	GCUAAA**UACUGU**AGGG-----
Ptr	-------------AAC**UACUGU**AUUCCCACAA-----------------------------------	GCUAAA**UACUGU**AGGG-----
Mml	-------------AAC**UACUGU**AUUCCCACAA-----------------------------------	GCUAAA**UACUGU**AGGG-----
Mmu	-------------AAC**UACUGU**AUUUCCACAA-----------------------------------	GCUAAA**UACUGU**AG---------
Cpo	-------------AAC**UACUGU**AUUCCCACGA-----------------------------------	GCUAAA**UACUGU**AG---------
Cfa	-------------AAC**UACUGU**AUUCCCACAA-----------------------------------	GCUAAA**UACUGU**AGAC-----
Fca	-------------AAC**UACUGU**AUUCCCACAA-----------------------------------	GCUAAA**UACUGU**AGAC-----
Eca	-------------AAC**UACUGU**AUUCCCACAA-----------------------------------	GCUAAA**UACUGU**AGAC-----

Supplementary Table 1

Supplementary Table 1. 3'-UTR Runx1 sequences across species. MREs for miR-144 are evidenced in bold. Hsa: Homo sapiens; Ptr: Pan troglodytes; Mml: Macaca mulatta; Mus musculus; Cpo: Cavia porcellus; Cfa: Canis familiaris; Fca: Felis catus; Eca: Equus caballus.
